# Tripeptidyl peptidase I promotes human endometrial epithelial cell adhesive capacity implying a role in receptivity

**DOI:** 10.1186/s12958-020-00682-0

**Published:** 2020-12-14

**Authors:** Leilani L. Santos, Cheuk Kwan Ling, Evdokia Dimitriadis

**Affiliations:** 1 Department of Obstetrics and Gynaecology, University of Melbourne, Parkville, VIC 3010 Australia; 2 Gynaecology Research Centre, The Royal Women’s Hospital, Level 7, 20 Flemington Road, Parkville, VIC 3052 Australia

**Keywords:** Endometrial receptivity, TPP1, SIRT1, BCL2, p53, Embryo implantation, Adhesion, Decidualization

## Abstract

The endometrium undergoes cyclic remodelling throughout the menstrual cycle in preparation for embryo implantation which occurs in a short window during the mid-secretory phase. It is during this short ‘receptive window’ that the endometrial luminal epithelium acquires adhesive capacity permitting blastocysts firm adhesion to the endometrium to establish pregnancy. Dysregulation in any of these steps can compromise embryo implantation resulting in implantation failure and infertility. Many factors contribute to these processes including TGF-β, LIF, IL-11 and proteases. Tripeptidyl peptidase 1 (TPP1) is a is a lysosomal serine-type protease however the contribution of the TPP1 to receptivity is unknown. We aimed to investigate the role of TPP1 in receptivity in humans.

In the current study, TPP1 was expressed in both epithelial and stromal compartments of the endometrium across the menstrual cycle. Expression was confined to the cytoplasm of luminal and glandular epithelial cells and stromal cells. Staining of mid-secretory endometrial tissues of women with normal fertility and primary unexplained infertility showed reduced immunostaining intensity of TPP1 in luminal epithelial cells of infertile tissues compared to fertile tissues. By contrast, TPP1 levels in glandular epithelial and stromal cells were comparable in both groups in the mid-secretory phase. Inhibition of TPP1 using siRNA compromised HTR8/SVneo (trophoblast cell line) spheroid adhesion on siRNA-transfected Ishikawa cells (endometrial epithelial cell line) in vitro. This impairment was associated with decreased sirtuin 1 (*SIRT1*), *BCL2 and p53* mRNA and unaltered*, CD44, CDH1, CDH2, ITGB3, VEGF A, OSTEOPONTIN, MDM2, CASP4, MCL1, MMP2, ARF6, SGK1, HOXA-10, LIF, and LIF receptor* gene expression between treatment groups. siRNA knockdown of TPP1 in primary human endometrial stromal cells did not affect decidualization nor the expression of decidualization markers prolactin (PRL) and insulin-like growth factor-binding protein 1 (IGFBP1). Taken together, our data strongly suggests a role for TPP1 in endometrial receptivity via its effects on epithelial cell adhesion and suggests reduced levels associated with unexplained infertility may contribute to implantation failure.

**Supplementary Information:**

The online version contains supplementary material available at 10.1186/s12958-020-00682-0.

## Background

The endometrium undergoes a coordinated cyclic remodelling during the menstrual cycle in preparation for embryo implantation. During a short ‘receptive period’ or ‘implantation window’ which occurs within the mid-secretory phase, the endometrium transforms from a non-receptive to an embryo-responsive state to allow for embryo implantation [1]. The natural fate of the receptive endometrium, in the absence of implantation, is development of a second set of changes that ultimately lead to menstruation [2, 3]. Dysregulation in any step during this process may cause abnormal remodelling of the endometrium leading to implantation failure and infertility.

Endometrial transformation during the implantation window is a result of the interplay between the ovarian steroid hormones, oestrogen and progesterone, the actions of which drive cellular and molecular mechanisms that operate in embryo implantation and development and the reproductive process [4–6]. The endometrial surface or luminal epithelium undergoes extensive changes to become adhesive to blastocysts [1] the mid-secretory phase or the implantation window thought to occur between ~days 19–23 of the cycle [1]. During the implantation window, the endometrial stromal cells which maximally express steroid hormone receptors differentiate to transform into decidualized stromal cells [1]. Endometrial stromal cells can act on the epithelial cells either by cell-cell or paracrine interactions during implantation [1]. Additionally, endometrial stromal cells isolated from women with infertility do not decidualize as effectively in vitro as stromal cells isolated from women with infertility [7]. Several cytokines, chemokines, growth factors, matrix metalloproteases and adhesion molecules all have important contributions to the implantation process as well as processes preceding it [8–12]. Information on lesser known proteases such as tripeptidyl peptidase I (TPP1) are lacking.

TPP1 is a lysosomal serine-type protease that sequentially removes tripeptides from the unmodified *N*-terminal of polypeptides and small proteins [13]. TPP1 is encoded by the *TPP1* gene [13] and is synthesized as an inactive 68-kDa proenzyme which is then processed in the lysosome into a mature 48-kDa form [14]. It is highly conserved and widely distributed in higher organisms and has a broad range of substrate specificity [15]. TPP1 has numerous functions one of which is digestion of various proteins in the lysosomes to release free amino acids and dipeptides which are then transported back to the cytoplasm for use in cell metabolism [14]. TPP1 has been associated with osteoclast degradation of bone [16], localised in the invasive front of tumours [14], positively correlated with breast cancer biomarkers cathepsin G, estrogen and progesterone receptors [17] and increased in other cancers such as thyroid adenocarcinoma and liver cancer [18]. Factors and processes operating in these pathologies overlap with that observed in endometrial receptivity and embryo implantation [19–21], thus supporting our rational for investigating the relevance and function of TPP1 in the above processes. We investigated the contribution of TPP1 to endometrial receptivity and embryo implantation via immunohistochemistry studies to localise TPP1 in the endometrium as well as in vitro epithelial cell adhesion and decidualization assays to explore its functional relevance.

We demonstrated that TPP1 immunoprotein is produced in human endometrial epithelial and stromal cells across the menstrual cycle. TPP1 staining intensity in endometrial luminal epithelial cells was reduced during receptivity in primary unexplained infertility. siRNA knockdown of TPP1 compromised Ishikawa cell line (receptive endometrial epithelial cell line) adhesive capacity for HTR8SVneo cell line (human trophoblast cell line) spheroids and reduced the expression of *SIRT1*, a known regulator of steroid hormone receptors. Gene expression of anti-apoptotic B-cell lymphoma 2 (*BCL-2*) and the tumour suppressor *p53* were similarly decreased in TPP1 siRNA-treated Ishikawa cells. By contrast, knockdown of TPP1 in primary human endometrial stromal cells (HESC) during decidualization had not effect on decidualization as measured by the effects on the expression of the decidualization markers, prolactin (*PRL*) and insulin like growth factor-binding protein 1 (*IGFBP1*). Our study suggests TPP1 promotes endometrial receptivity and embryo implantation via effects on endometrial epithelial cell adhesion and SIRT1, Bcl-2 and p53 stimulation and likely not via effects on endometrial stromal cell decidualization.

## Methods

### Ethics statement

Written informed consent was obtained from each patient and the study was approved by the Human Research Ethics Committee at the Royal Women’s Hospital (ID: #03066B).

### Primary endometrial tissue collection

Endometrial biopsies were collected by curettage from fertile women (26–42 years of age) with regular menstrual cycles (28–32 day cycles), during the proliferative (Days 6–13), early (Days 14–18), mid (Days 19–23) and late secretory phases (Days 24–28) of the menstrual cycle (*n* = 3–5/phase). Fertile women (28–40 years of age) had proven parity (> 1 parous pregnancy), no apparent endometrial abnormalities and were having surgery for Mirena insertion, benign ovarian cyst assessment or polypectomy. The endometrial tissue samples were collected by curettage and were from the functionalis layer. Women had regular menstrual cycles (27–32 days) and were not using pharmacological or intrauterine contraceptives and had not used hormones for at least 3 months prior to surgery. Additional samples were collected from women with primary unexplained infertility in the mid-secretory phase (*n* = 4; infertile). The infertility was defined as being unable to conceive for 1 year without the use of contraceptives and had no apparent endometrial dysfunction assessed by routine clinical investigations including hysteroscopic and laparoscopic diagnosis, including endometriosis, endometritis or other endometrial related disorders. Women with primary infertility were investigated for non-endometrial causes of their infertility, for example, ovarian dysfunction and tubal patency which were also used as exclusion criteria.

Partners of the infertile group had normal sperm analysis including sperm counts, motility and morphology. Mid-secretory phase was defined as days 19–23 of an idealized 28-day menstrual cycle. Samples collected were examined by gynaecological pathologists to confirm cycle stage and the absence of any histological abnormalities.

### Immunohistochemistry

Immunohistochemistry for TPP1 was performed on formalin-fixed endometrial tissue as described [12]. Fixed 4 μm sections on tissue slides were rehydrated and then antigen retrieval performed by microwave heating in 10 mM sodium citrate buffer for 5 min. Endogenous peroxidase activity was quenched in 3% hydrogen peroxide in methanol for 15 min. Sections were then incubated sequentially with nonimmune block (10% normal goat serum, 2% normal human serum in TBS; 30 min at RT), anti-human TPP1 primary antibody (0.14 μg/ml, #ab54685, Abcam, Cambridge, UK) or isotype negative control (Rabbit IgG, 0.14 μg/ml, #X0903, DAKO, Denmark) nonimmune block overnight at 4 °C) then finally with avidin/biotin reagents (Vectastain ABC Elite kit; Vector Laboratories, CA, USA) as per manufacturer’s instructions. Colour was developed with peroxidase substrate 3,3′-diaminobenzidine (DAB, DAKO) and sections counter-stained with haematoxylin (Sigma). Sections were dehydrated and mounted with coverslips using DPX (Sigma). Images were taken with an Olympus light microscope**.**

Staining intensity scores were determined by two individual scorers blinded to the patient characteristic and cycle stage of each section, as previously described [12]. Briefly, scores were given to each tissue type (luminal epithelium, glandular epithelium, stroma) on a scale of 0–3 (0: no stain, 3: strong stain).

### Cell lines

Ishikawa endometrial epithelial cells were provided by Dr. M. Nishida (Tsukuba University, Tochigi, Japan) and cultured in Dulbecco’s modified Eagle’s medium (DMEM) with 10% fetal calf serum (FCS; Gibco). Ishikawa cells are receptive endometrial epithelial cells possessing apical adhesiveness, as such are commonly used to study endometrial receptivity [12, 22] as it has similar characteristics to endometrial luminal and glandular epithelium, including the expression of steroid hormone receptors and possess apical cell adhesiveness [23]. The HTR8/SVneo trophoblast cell line (CRL-3271) exhibits features of invasive trophoblast cells, such as human leukocyte antigen-G (extravillous trophoblast marker) and cytokeratin-7 expression [23]. These cells were cultivated and maintained in Roswell Park Memorial Institute medium (RPMI) 1640 (Sigma-Aldrich) supplemented with 10% FCS.

### siRNA transfection of Ishikawa cells

Ishikawa cells (70–80% confluence) were transfected with TPP1 siRNA (10 nM, based on a concentration response) (Dharmacon, Lafayette, CO, USA) or scramble control (50 nM, Dharmacon) using Lipofectamine RNAiMAX transfection reagent (Thermo Fisher, Waltham, MA, USA) in Opti-MEM media (Thermo Fisher) according to the manufacturer’s instructions. After 24 h the transfection medium was replaced with fresh culture medium and Ishikawa cells were cultured for 48 h before being subjected to spheroid adhesion assay or other downstream analyses.

### HTR8/SVneo spheroid adhesion assay

Spheroids were formed using HTR8/SVneo cells (2000 cells/spheroid) in an ultra-low adhesion U-shaped 96 well plate (Corning, NY, USA) and cultured for 48 h at 37 °C. S Spheroids were harvested and transferred to transfected Ishikawa cell monolayer (20 spheroids/well of 96-well plate). Spheroid number per well was counted and recorded using a light microscope before incubation at 37 °C for 2 h. Following incubation, wells were gently washed with serum-free DMEM/F12 to remove non-adherent spheroids. The remaining spheroids were counted, and a percentage of adhered spheroids calculated [12, 24]. For qPCR analysis, Ishikawa cells were washed to remove spheroids then collected in TRI reagent for RNA extraction.

### Primary human endometrial stromal cell isolation and culture

Primary human endometrial stromal cells were isolated from patient endometrial biopsies as previously described [12, 24] with modifications. Briefly, endometrial tissue was subjected to collagenase (#CLS-3; Worthington Biochemical Corp, NJ, USA) digestion and filtration through 45- and 11-mm nylon meshes. Stromal cells contained in the filtrate were washed then resuspended in HESC media (DMEM/F12 (Sigma), 10% charcoal-stripped (cs) FCS (Gibco), 1% antibiotics (Sigma)) and adhered for 1–2 h to remove non-HESC contaminants. This method results in a 97% pure stromal cell culture [25]. Stromal cells were maintained in HESC media until required.

### Primary human endometrial stromal cell decidualization assay

Endometrial stromal cells were passaged into 6- and 12-well plates and grown to 70% confluency prior to siRNA transfection. Cells were transfected with TPP1 siRNA or scramble control (both 10 nM) for 48 h until they reached confluence. After transfection cells were cultured in decidualisation media (DMEM/F12, 2% csFCS, 1% P/S) containing 10^− 8^ M estrogen (Oestradiol; Sigma) and 10^− 7^ M methoxyprogesterone acetate (MPA; Sigma) or only 10^− 8^ estrogen as control. Decidualization media were replaced every 2–3 days and decidualization maintained until day 12 when cells were trypsinised and collected in 1 ml TRI reagent for RNA extraction. Decidualization was assessed by observation of characteristic cell morphology and measurement of PRL and IGFBP1 expression.

### RNA isolation and qPCR

Ishikawa cells and HESC were lysed with 1 ml TRI Reagent (Sigma) and RNA extracted according to the manufacturer’s protocol (Qiagen; MD, USA). Genomic DNA contamination was removed by treatment with TURBO DNA-free Kit (Thermo Fisher #AM1907) and RNA concentration determined in a Nanodrop spectrophotometer (Nanodrop 2000, Thermo). For RT-qPCR, 300 ng total RNA was converted to cDNA using SuperScript™ III First-Strand Synthesis System (18080–051, Thermo). PCR was performed on the Applied Biosystems ViiA7 system using SYBR Green Master Mix (#4367659, Thermo) as follows: 95 °C for 10 min and 40 cycles of 95 °C for 15 s followed by 60 °C for 1 min using relevant primers (Supplementary Table 1). Gene expression was normalized to *18S*. Relative expression levels were calculated using the comparative cycle threshold method (ΔΔCt).

### Statistical analysis

Statistical analysis was performed on original data using GraphPad Prism 8.0 and student’s t-test or one-way ANOVA as appropriate with a significance threshold of *P* < 0.05. Graphical data were presented as the mean ± SEM.

## Results

### TPP1 is abnormally reduced in the endometrial luminal epithelium in infertile women

**Fig. 1:**
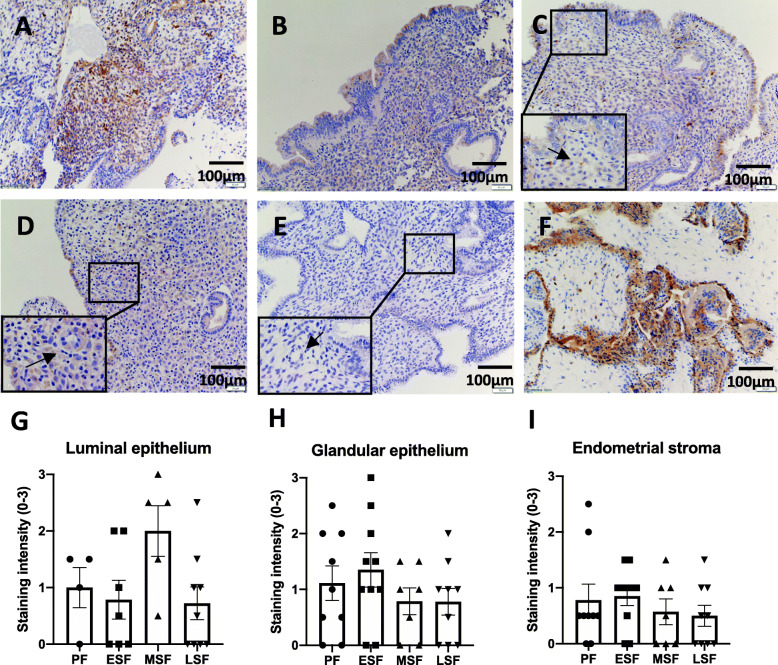
Immunohistochemistry staining of TPP1 in fertile human endometrial tissues across the cycle. **a** Proliferative phase, **b** early secretory phase, **c** mid-secretory phase, **d** late secretory phase, **e** mid-secretory phase probed with isotype-matched negative control, **f** human placental tissue positive control probed with TPP1. Immunohistochemistry staining intensity scores (mean ± SEM) of TPP1 expression in (**g**) luminal epithelial cells, (**h**) glandular epithelial cells and (**i**) stromal cells. PF = proliferative fertile, ESF = early-secretory fertile, MSF = mid-secretory fertile, LSF = late-secretory fertile, Scale bar = 100 μm. Unpaired student t-test were used with statistical threshold of *p* < 0.05

We immunolocalised TPP1 in normal endometrial tissues across all phases of the menstrual cycle as well as in the mid-secretory phase endometrium of women with primary unexplained infertility. Diffused cytosolic TIPP1 was observed in both stromal and epithelial compartments of the endometrium from all phases (Fig. 1a-d). There were TPP1-positive cells in spiral arteries and its surrounds. There was no staining observed with isotype control-incubated tissues (Fig. 1e) while strong and specific positive stain was observed in positive control placental tissue (Fig. 1f). TPP1 production was comparable across the menstrual cycle as shown by similar staining intensity scores in luminal (Fig. 1g) and glandular epithelium (Fig. 1h) and stroma (Fig. 1i).

**Fig. 2:**
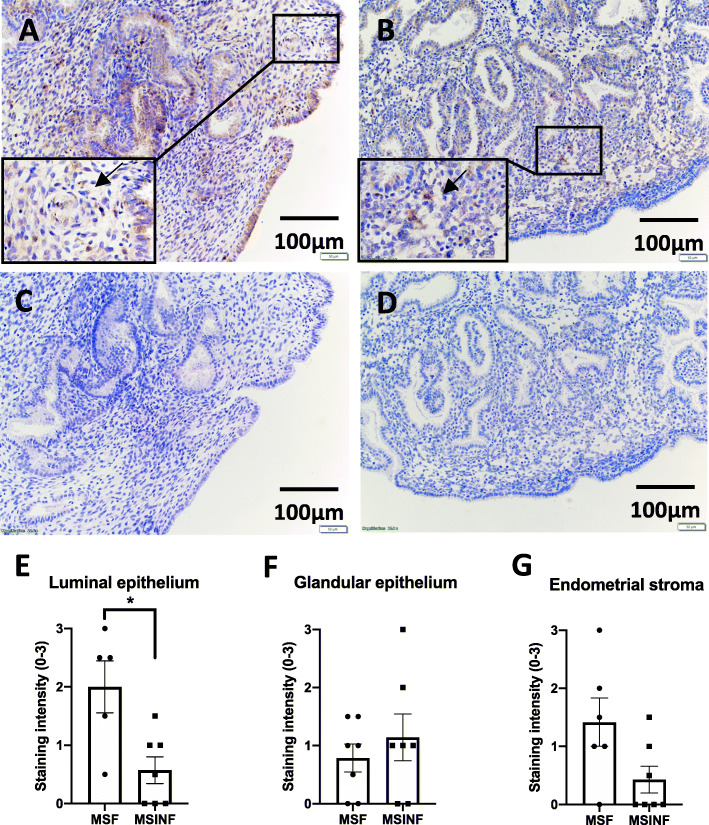
Immunohistochemistry staining of TPP1. Mid-secretory (**a**) fertile and (**b**) primary infertile human endometrial tissues, (**c**) anti-mouse isotype negative control, (**d**) human placental tissue positive control. Immunohistochemistry staining intensity scores (mean ± SEM) of TPP1 expression in (**e**) luminal epithelial cells, (**f**) glandular epithelial cells and (**g**) stromal cells. MSF = mid-secretory fertile, MSINF = mid-secretory infertile. Scale bar = 100 μm. Unpaired student t-test were used with statistical threshold of *p* < 0.05. *p < 0.05

Compared to fertile tissues (Fig. 2a), mid-secretory tissues from women with infertility exhibited reduced staining intensity of TPP1 (Fig. 2b and e) specifically in luminal epithelial cells. No staining was observed in isotype control-incubated tissues (Fig. 2c and d). TPP1 staining intensity scores in the endometrial glandular epithelium did not change between fertile and infertile tissues during receptivity cells (Fig. 2f). Similarly, TPP1 immunostaining intensity was not significantly different between infertile and fertile endometrium in the stroma (Fig. 2g).

### TPP1 siRNA knockdown impaired endometrial epithelial cell adhesive capacity and SIRT1, p53 and BCL2 gene expression

**Fig. 3:**
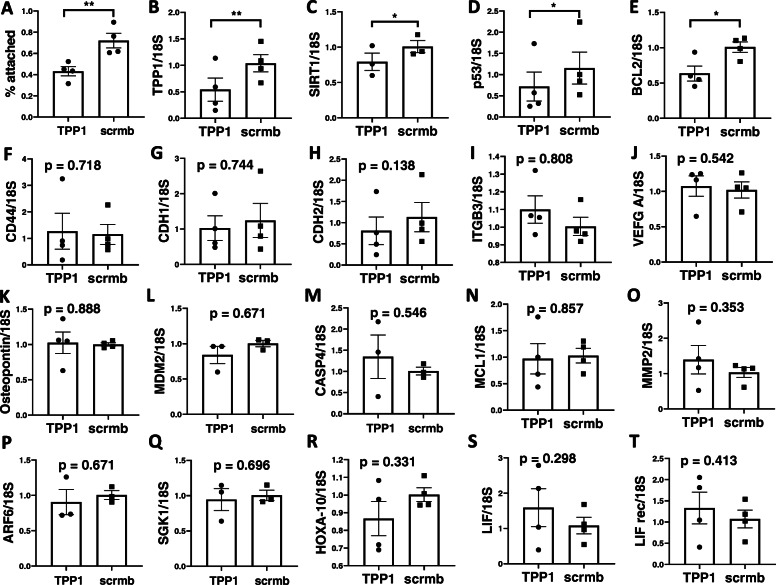
Effect of TPP1 siRNA knockdown on epithelial cell adhesive capacity and gene expression. **a** Percentage of adhered HTR8 spheroids on TPP1 siRNA or scrambled control siRNA transfected-Ishikawa cells after 4 h of incubation. **b** Confirmation of TPP1 siRNA knockdown efficiency by qPCR. Target gene expression in TPP1 siRNA-transfected Ishikawa cells (**c-t**). Analysis via paired student t-test with statistical threshold of *p* < 0.05. * *p* < 0.05, ** *p* < 0.01

To investigate the functional relevance of TPP1 on endometrial receptivity, an epithelial cell-spheroid cell adhesion assay was performed using TPP1 siRNA-transfected Ishikawa epithelial cell lines and HTR8/SVneo spheroids (Fig. 3). Ishikawa cells transfected with TPP1 siRNA had significantly reduced spheroid adhesion compared to scrambled control (Fig. 3a, *P* < 0.01). This reduction was associated with significant TPP1 knock down by 54 ± 12% (Fig. 3b, *N* = 4, *P* < 0.01) and reduced *SIRT1* (Fig. 3c, *P* < 0.05), *p53* (Fig. 3d, *P* < 0.05) and *BCL2* gene expression (Fig. 3e, *P* < 0.05). TPP1 knockdown in Ishikawa cells had no effect on the expression of *CD44, CDH1, CDH2, ITGB3, VEGF A, OSTEOPONTIN, MDM2, CASP4, MCL1, MMP2, ARF6, SGK1, HOXA-10, LIF,* and *LIF receptor* genes*.*

### TPP1 did not affect the expression of decidualization markers and primary human endometrial stromal cell morphology during decidualization

**Fig. 4:**
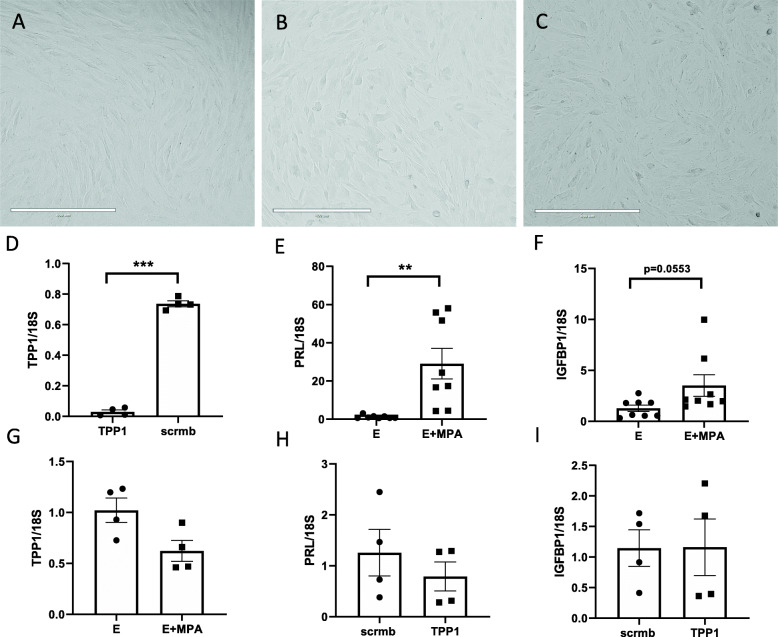
Effect of TPP1 siRNA knockdown on stromal cell decidualization and gene expression. Day 12 decidualization of HESC treated with either (**a**) estrogen only, (**b**) estrogen with MPA, or (**c**) estrogen with MPA and TPP1 siRNA transfection. HESC were treated with estrogen or estrogen with MPA (E + MPA) for 12 days before measuring TPP1 RNA and siRNA knockdown efficiency confirmed by qPCR (**d**). Effect of E + MPA on expression of PRL (**e**), IGFBP1 (**f**) and TPP1 (**g**). Expression of decidualization markers PRL (**h**) and IGFBP1 (**i**) were compared between E + MPA treated HESC that has been transfected with either TPP1 siRNA or a scrambled (scrmb) siRNA control. Scale bar = 400 μm. Analysis via paired student t-test or paired ANOVA test with significant threshold of *p* < 0.05. * = *p* < 0.05, ** = *p* < 0.01, *** = *p* < 0.001

We next sought to determine the function of TPP1 in endometrial stromal cell decidualization. Treatment of primary human endometrial stromal cells with estrogen and MPA for 12 days resulted in decidualization as indicated by a change from characteristic elongated fibroblast-shaped stromal cells (Fig. 4a) into a more rounded morphology characteristic of decidualized stromal cells (Fig. 4b). TPP1 siRNA-transfected HESC did not affect the morphology of HESC during decidualization in response to estrogen and MPA (Fig. 4c), despite sufficient TPP1 knockdown by 95 ± 1% (Fig. 4d, *N* = 4, *P* < 0.001) compared to scrambled siRNA control. Moreover, estrogen and MPA significantly induced the expression of the decidualization marker prolactin (*PRL*) (*P* < 0.05, Fig. 4e) and while the expression of *IGFBP1* did not reach significance compared to estrogen non-decidualized control treatment (Fig. 4f, *P* = 0.0553). Estrogen and MPA treatment of HESC did not significantly alter *TPP1* expression compared to estrogen treatment alone after 12 days of culture (Fig. 4g). Lastly, siRNA knockdown of endogenous TPP1 in HESC did not alter *PRL* (Fig. 4h) and *IGFBP1* (Fig. 4i) expression compared to estrogen and MPA treated decidualization control cells.

## Discussion

In this study, we report for the first time the endometrial expression of TPP1 across all phases of the menstrual cycle in both stromal and epithelial compartments of the endometrium. More importantly, compared to mid-secretory fertile endometrial tissues, infertile tissues exhibited significantly reduced TPP1 immunostaining intensity in the luminal epithelium. Targeted siRNA-mediated knockdown of *TPP1* in Ishikawa cells resulted in reduced HTR8-spheroid adhesion which was associated with upregulation of Ishikawa cell *SIRT1, BCL2 and p53* mRNA. By contrast, *TPP1* knockdown in primary HESC did not affect decidualization. While TPP1 localised to endometrial stromal cells in human endometrium, *TPP1* expression levels were not altered during decidualization in vitro.

TPP1 is a lysosomal aminopeptidase and is synthesized as an inactive proenzyme or zymogen that undergoes limited proteolysis to realize enzyme activity [14]. With a broad range of substrate specificity, the catalytic activity of TPP1 involves removal of tripeptides from an unmodified *N*-terminus of small proteins and polypeptides [13]. Human TPP1 is an integral part of the lysosomal proteolytic apparatus that includes hydrolytic enzymes such as cysteine, serine and aspartic proteases [14]. These enzymes are responsible for the digestion of proteins that are transported to the lysosomes [26].

TPP1 is widely expressed in various tissues and organs including bone marrow, placenta, lung, pineal, and liver [18, 26]. Under pathological conditions, TPP1 immunoreactivity has been demonstrated in neurological disorders, lysosomal storage diseases, inflammation and differentiated neoplasms [18, 26]. TPP1 protein has also been implicated in bone resorption involved in the degradation of bone collagen [16]. Although widely distributed, TPP1 appears to have a predilection for specific cell types. In the current study, TPP1 was widely expressed in the fertile endometrium across the menstrual cycle but was reduced in mid-secretory infertile tissues only in the luminal epithelium, conferring a likely location- and cell-specific function for TPP1. This is in keeping with reports that TPP1 is highly expressed in cells involved in peptide-hormone production or phagocytic function such as in epithelial cells of thyroids [18]. The extensive distribution of TPP1 in human tissues and its activity in various pathological conditions, imply that TPP1 activity is critical for efficient protein degradation in lysosomes of many cell types. In support, protease activity is important in the development of a receptive endometrium supporting its wide distribution demonstrated in this study [1]. Immunohistochemistry studies report TPP1 localized mainly in the invasive front of tumours where it acts as a matrix protease [26]. TPP1 participates in degradation of collagen and possibly other matrix protein [16]. Matrix protein breakdown is an important process in endometrial remodelling [27] suggesting this activity as a possible mechanism of action of TPP1 in the processes stated above.

An overlap in factors and processes involved in tumour invasion and blastocyst invasion of the receptive endometrium has been suggested [20, 21]. For a cell to invade it must adhere, de-adhere, migrate, degrade extracellular matrix to invade, therefore adhesion is a major process facilitating cell invasion. We report herein the reduction of Ishikawa cell adhesive capacity following knockdown of TPP1 suggesting it acts at the very early stages of implantation. This suggests that normal levels of TPP1 in the endometrial epithelium facilitates adhesion and migration of blastocysts to the endometrium.

TPP1 was produced by human endometrial stromal cells in the endometrium during the mid stromal cells [1]. Positive immunostaining was found in endometrial stromal cells surrounding the spiral arteries resembling decidualized endometrial stromal cells, where decidualization of endometrial stromal cells,is thought to be initiated [1]. We sought to determine whether blocking endogenous TPP1 altered decidualization in vitro. However, knockdown of TPP1 in primary HESC did not affect decidualization as measured by PRL expression a commonly used marker of decidualization. It is possible that other decidual markers may have been affected following knockdown of TPP1 during decidualization, or that other processes such as effects on endometrial stromal cell survival may have been affected. This remains to be determined in future studies. We also found in our culture model that TPP1 was not significantly increased following treatment with estrogen and MPA which induced decidualization markers. Previous studies have demonstrated that decidualization of HESC in vitro can be induced by estrogen and MPA over a 12 day period and also treatment with cAMP [28]. It is a possibility that TPP1 expression in HESC may require cAMP treatment to induce its expression during decidualization as has been previously demonstrated for other factors such as interleukin 11 [25].

Mechanistically, we demonstrated that TPP1 may act to regulate human endometrial cell adhesion via the alteration of *SIRT1*. SIRT1 is an enzyme that deacetylates proteins that contributes to cellular regulation [29]. Sirtuin proteins are increasingly implicated in the regulation of steroid hormone receptor activity suggesting it may be involved in decidualization, the endometrial cell types that maximally expresses progesterone-receptor during receptivity [29]. Sirtuin 1 inhibits ligand-independent activation of estrogen receptor, regulate nucleo-cytoplasmic shuttling of the progesterone receptor while inducing slow versus rapid response genes [29]. While TPP1 knockdown did not alter decidualization of human endometrial stromal cells that express high levels of progesterone receptor, it may affect their survival or genes expressed by the cells including sirtuins however this remains to be determined.

Other activities of SIRT1 that may be relevant to the contribution of TPP1 to endometrial receptivity and embryo implantation include regulation of tumour cell apoptosis [30], association with the tumour suppressor protein p53 [31] and its direct activation of E-cadherin in Ishikawa cells [32]. SIRT1 expression is reported to be unchanged in all phases during the normal menstrual cycle [33]. However, in Ishikawa cells forced overexpression of SIRT1 increased their adhesion while knockdown reduced their adhesive capacity [32] supporting our study that TPP1 may alter receptivity via *SIRT1***.**

Furthermore, TPP1 knockdown in Ishikawa cells reduced the expression of the anti-apoptotic B-cell lymphoma 2 (Bcl-2) the cells. Bcl-2 is potent regulator of apoptosis and plays a pro-survival role in various cell types [34]. Well known for its tumour suppressive properties, additional alternate roles in maternal reproduction has been shown for p53 [24], another factor reduced following TPP1 knockdown in Ishikawa cells. p53 is involved in a negative feed-back loop with leukemia inhibitory factor (LIF), a key regulator of endometrial receptivity and implantation [35]. LIF expression is stimulated by p53 via direct binding to the molecule’s promoter and LIF in turn negatively regulates p53 by phosphorylation of STAT3 which activates other downstream targets causing activation of a negative regulator of p53 [36]. This interaction suggests a pro implantation role for p53. While the Ishikawa cells are a commonly used cell line to model endometrial receptivity studies, studies in primary endometrial epithelial cells are required to confirm this data.

By contrast, the expression of other genes strongly implicated in endometrial receptivity and adhesive capacity including *CASP4* [37]*, MMP2* [38]*, SGK1* [39]*, ARF6* [40]*, CD44* [41]*, CDH1* [42]*, CDH2* [42], and *MDM2* remained unchanged in *TPP1* siRNA-transfected Ishikawa cells, suggesting TPP1 does not act via these molecules in exerting its effects on epithelial cells and receptivity associated adhesive capacity.

## Conclusion

This study identified that TPP1 likely contributes to endometrial receptivity and blastocyst attachment to the endometrial surface or luminal epithelium at least in part, via effects on *SIRT1*, *p53* and *BCL-2* expression, genes associated with cell survival. Collectively, while these data warrant further exploration of the exact contribution of TPP1 in endometrial receptivity and implantation in vivo, it suggests that reduced levels of TPP1 in endometrial epithelium contribute to implantation failure.

## Supplementary Information

**Additional file 1: SupplementaryTable 1.** Primers.

## Data Availability

All data generated through this study are included in this article.

## Abbreviations

Bcl-2: B-cell lymphoma 2
HESC: Human endometrial stromal cell
IGFBP1: Insulin-like growth factor-binding protein 1
PRL: Prolactin
SIRT1: Sirtuin 1
TPP1: Tripeptidyl peptidase 1
