# Supplementary material for: Tripeptidyl peptidase I promotes human endometrial epithelial cell adhesive capacity implying a role in receptivity
Source: Reprod Biol Endocrinol. 2020 Dec 14;18:124. doi: 10.1186/s12958-020-00682-0 (PMC7734757; doi:10.1186/s12958-020-00682-0)
Supplement: Supplementary file 1 — Additional file 1: SupplementaryTable 1. Primers. [file 12958_2020_682_MOESM1_ESM.docx]

**SupplementaryTable 1** Primers

| **Primer name** | **Primer sequence (5’ – 3’)** |
| --- | --- |
| *ARF6 forward* | GGAAACTTGAAACCCTCATG |
| *ARF6 reverse* | GGAAACTTGAAACCCTCATG |
| *BCL2 forward* | GATTGTGGCCTTCTTTGAG |
| *BCL2 reverse* | GTTCCACAAAGGCATCC |
| *CASP4 forward* | GGCAGGACAAATGCTTCTTC |
| *CASP4 reverse* | TGCGGTTGTTTCTCTCCTTT |
| *CD44 forward* | CTGCCGCTTTGCAGGTGTA |
| *CD44 reverse* | CATTGTGGGCAAGGTGCTATT |
| *CDH1 (E-cadherin) forward* | CAGGTCTCCTCTTGGCTCTG |
| *CDH1 (E-cadherin) reverse* | ACACCATCTGTGCCCACTTT |
| *CDH2 (N-cadherin) forward* | AGCCAACCTTAACTGAGGAGT |
| *CDH2 (N-cadherin) reverse* | GGCAAGTTGATTGGAGGGATG |
| *HOXA-10 forward* | CAGCAGAGGAGAAAGAGCGGC |
| *HOXA-10 reverse* | TTCCTGGGCAGAGCCTGAAG |
| *IGFBP1 forward* | TTTTATCACAGCAGACAGTG |
| *IGFBP1 reverse* | AATATATCTGGCAGTTGGGG |
| *ITGB3 forward* | ACTGCCTGTGTGACTCCGACT |
| *ITGB3 reverse* | CGCGTGGTACAGTTGCAGTAG |
| *LIF forward* | TGAACCAGATCAGGAGCCAAC |
| *LIF reverse* | CCACATAGCTTGTCCAGGTTG |
| *LIF rec forward* | CCTGGTAAATGCCAAGAAAG |
| *LIF rec reverse* | AAGTTTATCCCCATACTCCTAC |
| *MCL-1 foward* | TAGTTAAACAAAGAGGCTGG |
| *MCL-1 reverse* | ATAAACTGGTTTTGGTGGTG |
| *MDM2 foward* | CAGCAGGAATCATCGGACTCA |
| *MDM2 reverse* | ACACAGAGCCAGGCTTTCAT |
| *MMP2 forward* | GTGATCTTGACCAGAATACC |
| *MMP2 reverse* | GCCAATGATCCTGTATGTG |
| *Osteopontin forward* | ACAGCCAGGACTCCATTGAC |
| *Osteopontin reverse* | ACACTATCACCTCGGCCATC |
| *p53 forward* | CCCAAGCAATGGATGATTTGA |
| *p53 reverse* | GGCATTCTGGGAGCTTCATCT |
| *PRL forward* | TGACCCTTCGAGACCTGTTTG |
| *PRL reverse* | CTTGCTCCTTGTCTTCGGG |
| *SGK1 forward* | AGACTACATTAATGGTGGAGA |
| *SGK1 reverse* | ATTTCAGCAGCATAGAAACG |
| *SIRT forward* | AAGTTCCATACCCCATGAAG |
| *SIRT reverse* | ATTCACCACCTAACCTATGAC |
| *TPP1 forward* | CCTCCACACGGTGCAAAAATG |
| *TPP1 reverse* | CTCTGCTTGTCGGATGCTCAG |
| *VEGFA forward* | AATGTGAATGCAGACCAAAG |
| *VEGFA reverse* | GACTTATACCGGGATTTCTTG |
